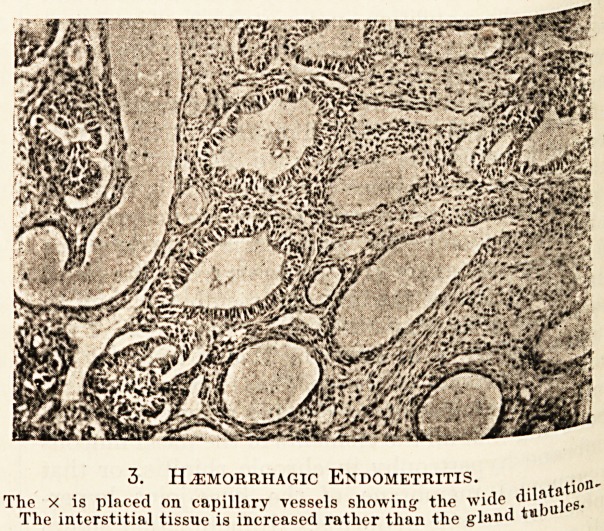# Endometritis and the Operation of Curettage

**Published:** 1908-01-25

**Authors:** Thos. G. Stevens

**Affiliations:** Physician, Hospital for Women, Soho Square; Obstetric Tutor, St. Mary's Hospital, Paddington.


### Hospital Clinics.

### ENDOMETRITIS AND THE OPERATION OF CURETTAGE.

By THOS. G. STEVENS, M.D., B.S.Lond., F.R.C.S.Eng., M.R.C.P.Lond.; Physician, Hospital for Women, Soho Square; Obstetric Tutor, St. Mary's Hospital, Paddington.

Paper read before the Norwich Medico-Chirurgical Society, October 1, 1907.

Endometritis is even now a disease around which discussions take place, and at least one writer in a recent edition confesses to not knowing what it is or how it arises. This is truly going back to the days of Matthews Duncan, and is not quite fair to the band of pathologists who have done a considerable amount of work on the subject, and thrown not a little light upon it. The great difficulty we are confronted with is to recognise what is pathological in the endometrium, and what is the result of pre-menstrual change in the mucous membrane. Strictly speaking, we do not yet know what is the real structure of the normal endometrium of an adult and its exact changes during the menstrual cycle. We do know this, however, that in the majority of cases of menorrhagia in which no gross lesion can be found, the endometrium is more or less thickened and the glands altered; whereas we find no thickening of the mucous membrane in many cases we curette, where menorrhagia is absent, such as simple spasmodic dysmenorrhœa in virgins. If a thickened endometrium is a part of the menstrual cycle, we should find it alike in women who have or who have not menorrhagia.

Are we justified, on the other hand, in considering this thickening to be the result of inflammation, and in calling the condition endometritis? The latest observers on this point, Hitschmann for one, in a recent paper, say that the only true form of endometritis is that in which the connective tissue alone is affected, namely interstitial endometritis. If this is so, endometritis is a very rare disease; but personally I cannot convince myself that it is true. I am persuaded that the common forms of endometritis, known as endometritis glandularis hypertrophica and hyperplastica, are in many cases really the result of inflammation of the mucous membrane, and in others arise after long-continued congestion. I see no more difficulty in believing that a hypertrophy of all the elements of the endometrium occurs as a result of inflammation or chronic congestion, than in believing that all the elements of the nasal mucous membrane hypertrophy in chronic rhinitis, or that the synovial membrane of the knee-joint hypertrophies in chronic synovitis. Inflammation shows itself everywhere by two factors, namely diapedesis of leucocytes and overgrowth of the tissues of a part. In acute inflammations the former greatly overrides the latter, but in chronic inflammatory processes overgrowth may be the only tangible result, as we see in the nose and in the knee-joint. For these reasons I have acquired the habit of regarding the common forms of endometritis as the overgrowth moiety of inflammation of the endometrium, or a similar result following long-continued congestion of the organ without real inflammation.

We must all be familiar with cases of bacterial infection of the uterus, at first acute, and then settling down to a chronic condition accompanied by menorrhagia, in which eventually we find the mucous membrane thickened but not obviously inflamed in the leucocytic sense. In such thickened mucous membranes we cannot demonstrate micro-organisms, because they have died out; neither, however, can we always demonstrate micro-organisms in chronically inflamed joints or in long-standing cases of pyosalpinx, though the original cause may have been undoubtedly bacterial.

The structure of the thickened endometrium is variable, but usually conforms either to the hypertrophic or to the hyperplastic type. In the former we find great increase in size, length and tortuosity

1. Hypertrophic Endometritis.
Shows the very regular enlargement of the gland tubules, with corresponding increase in the interstitial tissue.

of the glands without any obvious increase in number, whilst in the latter we find increase in number of the glands and extreme complexity in their shape and arrangement. Whilst in the hypertrophic form the gland tubule in section is usually circular, in the hyperplastic it may be very irregular in outline. In both, however complex the gland tubules may be, the benign character of the lesion is shown by the arrangement of the epithelium, namely in a single columnar layer on a basement membrane. In both these forms the stroma is composed of young connective tissue, which may be termed "embryonic" for the want of a better name. This stroma is always very vascular, and sometimes the capillary vessels are so enlarged as to present an angiomatous appearance. In extreme cases of vascularity the glands may not be a very marked feature, and it is to this class of endometritis that the term "hæmorrhagic" has sometimes been applied. The surface of the endometrium in these cases is never quite smooth, but it may be so irregular that polypoid projections occur here and there, a condition to which the term endometritis polyposa has been given. The term

" fungoid " endometritis, though widely used, has no strict application, and should be dropped from the nomenclature. These minor distinctions do not alter the main classification into hypertrophic and hyperplastic forms. We have no knowledge at present why the one or the other of these forms appears in a given case, for the ætiology of both is apparently the same.

True interstitial endometritis is not a common condition, but when it occurs is usually the product of infection of a senile uterus. Here there is increase of fibrous tissue with gland destruction, and often the surface towards the uterine cavity is really inflamed and covered with granulations. It is seen most markedly in the cases of retention of pus in the uterus in elderly women, so often met with in association with carcinoma of the cervix or body.

2. HYPERPLASTIC ENDOMETRITIS.
Shows the very irregular shape of the gland tubule and overgrowth of all elements in the mucous membrane.

### SYMPTOMS.

Briefly stated, these are menorrhagia, pain and discharge. The menorrhagia is sometimes pure in type, but often a blood-stained discharge occurs between the periods. The patients, however, do not get " floodings " apart from menstruation, and manipulation of the uterus does not as a rule cause bleeding. The discharge is watery in character, very different to the " unboiled white of egg " discharge of cervical catarrh. As I have said, this discharge may be blood-stained, especially in the cases of great vascularity above mentioned. The pain is chiefly sacral in character, but is often referred to the ovarian regions, the ovaries no doubt sharing in a general pelvic congestion. The uterus is often tender to the touch, and it is said that the sound always causes pain on passing. As I never pass the sound, except as a preliminary to an operation, I do not vouch for this, and I cannot let this opportunity pass without suggesting to you that the sound should not be used for consulting-room diagnosis. The danger of carrying infection to the Fallopian tubes by the sound cannot be overestimated, not to speak of the occasional chance of producing abortion or perforating the uterus. The sound gives us no information which a trained pair of hands cannot find out by bi-manual examination.

### TREATMENT.

The treatment of endometritis in a word is curettage ; nothing else is likely to give any lasting benefit. The older methods of swabbing out with caustics on coated probes, and of injecting solutions into the uterus, are proved to be futile proceedings, and belong to the tinkering methods of twenty years ago; and they cannot be too strongly condemned. In the early stages, where congestion is the real lesion without any pronounced change in the endometrium, ergot sometimes does good if given in full doses, and in my experience no other drug can be relied upon to produce the same effect. When, however, the endometrium is thickened, ergot will not remove it, and curettage must be performed to cure the patient. We have no means of proving it, but no doubt many of these cases cure themselves by a process of atrophy after the menopause ; but as so many occur in middle-aged or even young women, the long wait for such a natural cure cannot be tolerated.

Of all gynæcological operations, that of curettage of the uterus is the most widely performed, and in cases where it has been performed for endometritis the results are sometimes disappointing. I feel quite certain of this, and have no doubt that cases are known to you all in which the operation has been done without any lasting benefit. In a large hospital out-patient department I often see cases which have been curetted without the slightest improvement. The fact is that uterine curettage, if it is to be curative in cases of endometritis, must be a serious proceeding performed with the strictest asepsis. Herein, to my mind, lies the only too common cause of failure to cure the disease. The operation in too many instances is performed without strict attention to aseptic ritual, and as a result the uterus is reinfected at once ; or rather remains infected, if the primary cause of the disease was bacterial, and is infected at the time of the operation if the endometritis was originally the result of simple congestion. This result is easily brought about by imperfect disinfection of the vagina or by instruments and fingers that are not sterile. I believe, too, that the after treatment is often responsible for reinfection of the uterus even if the operation was aseptic. It is usual to have the vagina

3. HÆMORRHAGIC ENDOMETRITIS.

The × is placed on capillary vessels showing the wide dilatation. The interstitial tissue is increased rather than the gland tubules.

douched daily for a few days after the operation, and there can be no surer method than this of infecting the vagina and uterus, unless performed with the same care as a surgical operation. In my practice vaginal douching after operations has been discarded for years, except after vaginal hysterectomy. I have never seen a case in which I have had cause to regret interdicting vaginal douches after operations. Infection by fingers ought to be always avoidable, because there is no necessity to touch the vagina or uterus with the fingers or anything that has been in contact with them. If the finger must be put in, a freshly boiled glove or finger stall should be used, as there is no known method by which the fingers can be rendered sterile for more than a few minutes.

With regard to the details of the operation I should like to mention a few points. First, shaving is necessary, or at least cutting the hair short with the smallest beard clipper. Next, to disinfect the vagina and vulva, douching is absolutely useless. They must be cleansed in the same way that skin is prepared, two solutions being the minimum required, one a solution of soap in ether, the other an efficient antiseptic such as 1 in 160 lysol solution. The soap solution is poured into the vagina and energetically scrubbed into every part with small sterilised-wool pledgets held in forceps. After about three minutes of this, the vagina and vulva are scrubbed with more wool and the lysol solution. At least three minutes should be employed in this proceeding. No vaginal douche will cleanse the folds like this method of scrubbing. After this disinfection nothing which has not been sterilised must be put into the vagina.

The dilatation of the uterus is best performed with metal Hegar's dilators, and two precautions must be observed, namely steady pressure well controlled to avoid suddenly perforating the uterus, and care to avoid splitting the musculature at the level of the internal os by dilating too much. This splitting occurs fairly frequently, and is not of much import if the operation is aseptic, and if dilatation is at once stopped. Further attempts at dilatation must lead to further splitting, and it has occurred fairly often that larger dilators have been pushed into the split instead of the uterus, a false passage, or even perforation resulting. The actual curettage must be thorough, great care and a sharp curette being required to remove the endometrium at the fundus. If any application is made after curettage (I nearly always use iodised phenol for this purpose—1 part of solid iodine to 5 parts of liquid carbolic acid) it must be applied on coated probes which have been previously boiled. To coat a probe in the middle of an operation with bare hands means infecting the wool used, and is not satisfactory, even with so powerful an antiseptic as iodised phenol. Before applying an antiseptic it is good to wash out any débris from the uterus with a small-sized Bozemann catheter, but this may be omitted if the uterus is mopped out with sterile wool on a holder.

It is usual to finish by plugging the vagina lightly with iodoform or other antiseptic gauze, but it must not be forgotten that no dry gauze is sterile, and therefore whatever is used ought to be soaked in a strong antiseptic for several days previously.

The gauze must be removed in twenty-four hours, and then nothing whatever should be done locally until the endometrium has re-formed; no douches should be given, and no examinations made. Although these details appear to be somewhat complicated, they constitute an aseptic technique as simple in character as that usually adopted for a laparotomy. When once this routine is mastered but little preparation is required, and the results of the operation performed in this manner are so infinitely superior that the trouble is worth taking.

Curettage is undertaken in two other conditions besides endometritis, namely retained products of conception after abortion or labour, and suspected cancer of the body of the uterus. The details of the operation are essentially the same, but let it not be forgotten that the great danger of the curette is here encountered. Perforation of the uterus by the curette *in utero* has occurred much more commonly in the subjects of recent pregnancy than in any others. It is wise, therefore, not to use a curette at all for removing products of conception, unless the uterus is partly involuted. For recent conditions the finger is the safer instrument, and will often locate a small placental mass attached to the fundus that the curette might miss entirely. On the other hand, there is a growing tendency to use the blunt flushing curette for clearing out the *débris* of recent abortions, and it must be admitted that, done under anæsthesia with a contracted uterus, this affords a clean and certain method of emptying the uterus. But this proceeding must be looked upon as an operation requiring an anæsthetic and some experience of intra-uterine operations; it cannot be safely performed without anæsthesia by guiding the curette into the uterus along a finger. I know, however, that this is the way in which it is often done, and its risk is often but little appreciated. There is always some softening of the uterine wall in these cases, and if there is any inflammatory change a sound or curette will pass through with hardly any resistance at all.

When carcinoma of the body of the uterus is suspected the curette is the only means at our disposal to confirm the diagnosis. When cancer is present the sharp curette brings away thick masses of growth instead of the narrow gelatinous-looking ribbons of mucous membrane usually obtained in endometritis. The microscope must be used to make quite certain, and the proper preservation of curetted fragments is a very important part of the operation. They should be placed at once before they have had time to dry in either 70 per cent. alcohol or, better still, 10 per cent. formalin in normal saline solution. Either of these solutions gives good fixation, and the specimens can then be cut in paraffin. To a skilled pathologist there is not one case in fifty which presents any difficulty in the diagnosis, but operators will do well not to attempt this for themselves.

Let it be remembered that not even an expert can perform miracles; the specimen must be properly preserved, cut and stained, that the end may be satisfactory to all concerned.

It is well to note that curettage is not recommended for cases of acute septic endometritis of puerperal or gonorrhœal origin. Considerable controversy has taken place on this point, but it is generally agreed now that these cases are often made worse, and even fatalities caused, by curettage for these conditions. The reason for this is not far to seek. Acute endometritis, if there is any attempt at localisation of the lesion, is accompanied by an intense leucocytosis in the endometrium. If this layer of leucocytes is removed, a fresh area for infection is laid bare; when the organisms are of great virulence no further leucocytosis occurs, and a general infection may result. It is impossible in such cases to remove all the bacteria present, and so it is impossible to avoid reinfection of the endometrium. In treating such cases it is much wiser to dilate the cervix, wash out with an antiseptic douche, remove any retained material with the finger, and then plug the uterus with iodoform gauze. If such treatment is followed by any improvement there is no reason why it should not be repeated. Although a somewhat painful process, this can be done without an anæsthetic, using a speculum, and pulling down the uterus with a tenaculum. At the same time, if any signs of local pelvic peritonitis occur, it is good treatment at once to incise the posterior vaginal fornix, open Douglas' pouch, let out any fluid present, and then drain with gauze. The fluid in the pelvis in these cases is highly infective, and general peritonitis would be likely to occur if such a collection were opened by the abdominal route. Laparotomy should be reserved for cases of general peritonitis following acute septic endometritis.

## Figures and Tables

**1. f1:**
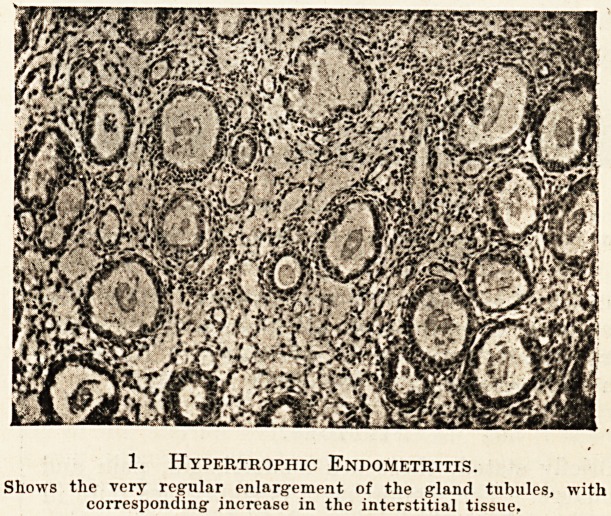


**2. f2:**
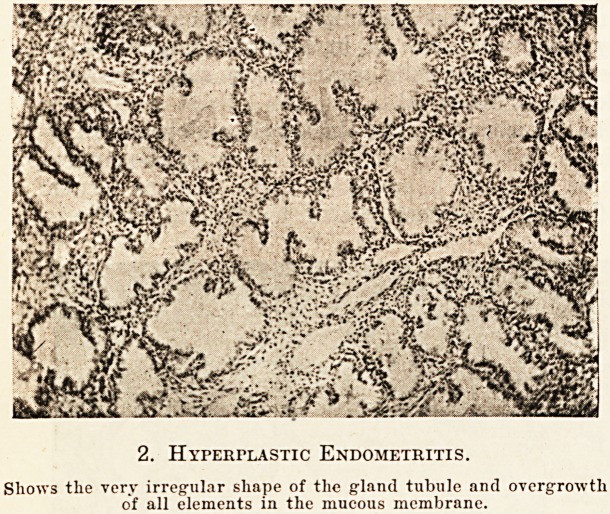


**3. f3:**